# Service Evaluation of COVID and Non-COVID Admission Trends to an East Midlands General Adolescent Psychiatric Inpatient Unit

**DOI:** 10.1192/bjo.2022.418

**Published:** 2022-06-20

**Authors:** Sue Fen Tan, Parveen Chand

**Affiliations:** Nottinghamshire Healthcare NHS Foundation Trust, Nottingham, United Kingdom

## Abstract

**Aims:**

To explore the differences in admissions between the first COVID-19 lockdown cohort and a pre-COVID-19 cohort.

**Methods:**

23 young people who were admitted to an East Midlands General Adolescent Inpatient Unit during the first COVID-19 lockdown from March 2020 to September 2020 were compared with the 48 young people who were admitted in the same period in 2019. Demographic details, admission duration and reasons, mental health act (MHA) status, diagnoses, functional status, and incidents were obtained retrospectively from the trust's online records.

**Results:**

The unit received more female admissions prior to lockdown (60.4% Vs 47.8%). Approximately 30% of adolescents in the pre-COVID-19 group were not in education whereas those admitted during COVID-19 were all receiving education. More of the pre-COVID-19 group attended school than college and more of the COVID-19 group were employed, consistent with a lower mean age of admission in the former group. Most of the COVID-19 admissions were local and none were out of area. Young people were also more likely to be looked after by their parents during COVID-19 (82.6%) and none were taken care of by their relatives.

Pre-COVID019 admissions were discharged sooner than their counterparts, which had 13% of admissions between 6–9 months. Both cohorts had mainly informal admissions due to risk to self. Most of the COVID-19 admissions were due to anxiety, followed by self-harm while the majority of pre-COVID-19 admissions were due to depression and PTSD. 43% of the COVID-19 admissions had at least one comorbid diagnosis, notably depression. More adolescents in the COVID-19 cohort were not started on any psychiatric medication during and after admission.

The mean number of incidents were two times higher in the COVID-19 group; self-harm was the most common reason. There was more violence towards staff during lockdown. However, absconsion, possession of contraband items, and staff error were higher in the pre-COVID-19 group.

**Conclusion:**

The introduction of COVID-19 restrictions was associated with a change in both the frequency and nature of inpatient admissions to this ward. Less young people were admitted during COVID-19, more frequently with anxiety as the primary reason and stayed for longer. Although the pre-COVID-19 group received more psychiatric medication, it is unclear if this contributed to a better functional status overall. This service evaluation also demonstrated the impact of COVID-19 on young people's mental health and life circumstances. An exploration of these trends in other units across the country would increase the generalisability of results.

